# Comparative diagnostic accuracy of laser fluorescence and conventional caries detection methods in children: a systematic review and meta analysis

**DOI:** 10.3389/fdmed.2026.1798996

**Published:** 2026-05-18

**Authors:** Pratyasha Sharma, Nishi Joshi, Srikala Bhandary

**Affiliations:** Department of Pediatric & Preventive Dentistry, AB Shetty Memorial Institute of Dental Sciences (ABSMIDS), NITTE (Deemed to be University), Mangalore, Karnataka, India

**Keywords:** dental caries, DIAGNOdent, diagnostic accuracy, laser fluorescence, meta-analysis, pediatric dentistry, systematic review

## Abstract

**Background:**

Early detection of dental caries in children is essential for implementing preventive and minimally invasive treatment strategies. Laser fluorescence–based devices have been proposed as adjunctive diagnostic tools; however, their diagnostic accuracy compared with conventional methods remains uncertain.

**Objective:**

To evaluate the diagnostic accuracy of laser fluorescence devices compared with conventional visual and radiographic methods for caries detection in pediatric populations.

**Methods:**

A systematic review was conducted following PRISMA 2020 guidelines and registered in PROSPERO (CRD420251274327). Electronic searches were performed in PubMed/MEDLINE, Scopus, Web of Science, and the Cochrane Library from inception to December 2025. Clinical studies involving children (<18 years) that evaluated laser fluorescence devices for caries detection were included. Risk of bias was assessed using QUADAS-2. Where sufficient data were available, quantitative synthesis was performed using bivariate random-effects and hierarchical summary receiver operating characteristic (HSROC) models.

**Results:**

Twenty-one studies were included in the qualitative synthesis. Quantitative meta-analysis was conducted for enamel-level (D1) and dentinal-level (D3) diagnostic thresholds. At the D1 threshold (8 studies), pooled sensitivity was 0.84 (95% CI 0.80–0.87) and specificity was 0.77 (95% CI 0.67–0.84), with an area under the curve (AUC) of 0.86. At the D3 threshold (10 studies), pooled sensitivity was 0.81 (95% CI 0.77–0.85) and specificity was 0.89 (95% CI 0.84–0.92), with an AUC of 0.81. Heterogeneity was influenced by lesion thresholds, dentition type, reference standards, and diagnostic protocols. Studies with higher risk of bias tended to report greater sensitivity estimates.

**Conclusion:**

Laser fluorescence devices demonstrate good sensitivity for detecting early carious lesions in children and may be useful adjuncts for preventive diagnosis and monitoring. However, variability in specificity and methodological heterogeneity across studies indicate that these devices should complement, rather than replace, conventional visual and radiographic examination.

**Systematic Review Registration:**

https://www.crd.york.ac.uk/PROSPERO/view/CRD420251274327, identifier CRD420251274327.

## Introduction

Dental caries remains the most prevalent chronic disease in childhood worldwide, affecting both primary and permanent dentitions and imposing a substantial burden on oral health–related quality of life. Early detection of carious lesions, particularly non-cavitated enamel lesions, is critical for implementing preventive and minimally invasive strategies that can arrest or reverse disease progression. Conventional diagnostic methods, including visual–tactile examination and bitewing radiography, continue to serve as the foundation of caries detection in clinical practice; however, these methods have well-recognized limitations, especially in detecting early enamel demineralization and proximal lesions in children (1–3).

Visual inspection, even when standardized systems such as the International Caries Detection and Assessment System (ICDAS-II) are employed, remains inherently subjective and dependent on examiner experience, surface conditions, and lesion activity (4). Radiographic examination, although valuable for proximal caries detection, is limited by its reduced sensitivity for early enamel changes and raises concerns related to cumulative radiation exposure, particularly in pediatric populations (5). These limitations have prompted the development and clinical adoption of adjunctive diagnostic technologies aimed at improving the early detection and objective assessment of carious lesions.

Laser fluorescence–based devices represent one such adjunctive approach and operate on the principle of detecting fluorescence emitted by bacterial metabolites, particularly porphyrins, within carious tooth structure (6). Devices such as DIAGNOdent and DIAGNOdent Pen have been evaluated for their diagnostic performance across different tooth surfaces and dentitions. Experimental and clinical studies have suggested that laser fluorescence may offer higher sensitivity for early enamel lesions compared with conventional methods, although concerns regarding specificity and false-positive readings remain (7–9). The diagnostic performance of these devices is influenced by multiple factors, including lesion depth, surface characteristics, plaque or staining, and diagnostic thresholds, which may contribute to variability across studies.

In pediatric dentistry, the diagnostic challenge is further compounded by limited patient cooperation, mixed dentition, and anatomical variations of primary teeth, which differ significantly from permanent teeth in enamel thickness and mineral content (10). These factors may influence the performance and reliability of laser fluorescence devices in children, necessitating age- and dentition-specific evaluation. Several clinical studies have assessed the diagnostic accuracy of laser fluorescence in children; however, reported results vary widely depending on study design, reference standards, lesion thresholds, and device type (11–13). Variability in reference standards, including histological validation, operative assessment, and combinations of clinical and radiographic methods, further complicates interpretation of diagnostic accuracy outcomes.

Although previous reviews have addressed caries detection technologies in general populations, there is a lack of focused synthesis specifically examining the diagnostic accuracy of laser fluorescence devices compared with conventional caries detection methods in pediatric populations. Furthermore, differences in lesion location (occlusal, proximal, smooth surfaces), dentition type, and diagnostic thresholds across studies make it difficult to draw definitive clinical conclusions regarding their utility in children. Therefore, the present systematic review aimed to evaluate and compare the diagnostic accuracy of laser fluorescence devices with conventional visual and radiographic methods for caries detection in pediatric populations, and to synthesise the available evidence to clarify their clinical utility and limitations.

## Materials and methods

Registration in the PROSPERO database was completed following the screening phase. (CRD420251274327). This systematic review was conducted according to the Preferred Reporting Items for Systematic Reviews and Meta-Analyses (PRISMA) 2020 (14) guidelines.

### Research question

In this systematic review, the aim was to assess and compare the diagnostic accuracy of laser fluorescence devices with conventional caries detection methods in children. The research question was structured using the Population–Index test–Reference standard–Target condition (PIRD) (15) framework:

Population (P): Children and adolescents (<18 years) with primary, permanent, or mixed dentition.

Index Test (I): Laser fluorescence–based caries detection using DIAGNOdent, DIAGNOdent Pen, and similar laser fluorescence-based optical devices.

Reference Standard (R): Histological validation, operative validation, temporary tooth separation, or combinations of clinical visual assessment and radiographic examination depending on study design.

Comparator: Conventional caries detection methods, including visual examination [e.g., International Caries Detection and Assessment System (ICDAS), visual–tactile assessment] and bitewing radiography.

Target Condition (D): Detection of dental caries at different stages (initial enamel lesions, non-cavitated lesions, dentinal lesions, and cavitated lesions) on occlusal, proximal, or smooth surfaces.

### Sources of data

An electronic systematic search of the literature was conducted across four major databases: PubMed/MEDLINE, Scopus, Web of Science, and the Cochrane Library. The databases were searched from inception to December 2025. In addition, a manual search of reference lists of included studies and relevant review articles was performed to identify potentially eligible publications. Manual search using Google search engine was also done.

The Embase database was not searched due to lack of institutional access; however, efforts were made to ensure comprehensive coverage through multiple database searches and manual screening.

### Search strategy

A comprehensive search strategy was developed for PubMed/MEDLINE, Scopus, Web of Science, and the Cochrane Library databases. Searches were conducted from database inception until December 2025. Controlled vocabulary terms (MeSH) and free-text keywords related to dental caries, laser fluorescence devices, and pediatric populations were combined using Boolean operators (AND, OR).

Search terms included variations of: “*dental caries,” “caries detection,” “caries diagnosis,” “laser fluorescence,” “DIAGNOdent,” “DIAGNOdent Pen,” “child,”* and “*adolescent.”* Reference lists of included articles were manually screened to identify additional relevant studies.

Only studies published in English were included. Duplicate records were identified and removed using Mendeley Reference Manager (Version 2.136.0).

The complete search strategies for each database are presented in Table 1 to ensure reproducibility.

**Table 1:** Detailed electronic search strategies for each database.

**Database**	**Search strategy**	**Results**
PubMed (MEDLINE)	((“DIAGNOdent”[All Fields] OR “DIAGNOdent Pen”[All Fields])) AND ((“Dental Caries”[MeSH Terms] OR (“dental”[All Fields] AND “caries”[All Fields]) OR “dental caries”[All Fields])) AND ((“caries detection”[All Fields] OR “caries diagnosis”[All Fields])) AND ((“Child”[MeSH Terms] OR “Adolescent”[MeSH Terms] OR child*[All Fields] OR pediatric*[All Fields] OR paediatric*[All Fields]))	140
Scopus	TITLE-ABS-KEY (“DIAGNOdent” OR “DIAGNOdent Pen”) AND (LIMIT-TO (EXACTKEYWORD, “Dental Caries”) OR LIMIT-TO (EXACTKEYWORD, “Caries Detection”)) AND (LIMIT-TO (EXACTKEYWORD, “Child”) OR LIMIT-TO (EXACTKEYWORD, “Adolescent”)) AND (LIMIT-TO (LANGUAGE, “English”))	122
Web of Science	TS = (“DIAGNOdent” OR “DIAGNOdent Pen”) AND TS = (“dental caries” OR “caries detection” OR “caries diagnosis”) AND TS = (child OR children OR adolescent)	65
Cochrane Library	((“DIAGNOdent” OR “DIAGNOdent Pen”) AND (“dental caries” OR “caries detection” OR “caries diagnosis”) AND (child OR children OR adolescent)):ti,ab,kw	68

### Eligibility criteria

The eligibility criteria were defined according to the Population–Index test–Reference standard–Target condition–Study design (PIRD-S) framework.

Only clinical studies involving pediatric participants (<18 years) were included. Studies were considered eligible if diagnostic assessments were performed *in vivo*, even when histological validation was subsequently conducted following tooth extraction. Studies conducted exclusively on extracted teeth without prior clinical assessment were excluded unless pediatric origin and clinical relevance were clearly specified. For studies including mixed-age populations, only data corresponding to pediatric participants were included where separable.

### Inclusion criteria

Population: Children and adolescents (<18 years) with primary, permanent, or mixed dentition.

Index Test: Laser fluorescence–based caries detection devices, including DIAGNOdent, DIAGNOdent Pen, and similar fluorescence-based systems.

Comparator: Conventional caries detection methods, including visual examination [e.g., International Caries Detection and Assessment System [ICDAS] (16), visual-tactile assessment], bitewing radiography, or other clinical diagnostic methods.

Reference Standard: Histological validation, operative validation, temporary tooth separation, or combinations of clinical and radiographic assessment depending on study design.

Target Condition: Detection of dental caries lesions at different stages (initial enamel lesions, non-cavitated lesions, dentinal lesions, or cavitated lesions) on occlusal, proximal, or smooth surfaces.

Outcomes: Studies reporting diagnostic accuracy measures, including sensitivity, specificity, predictive values, receiver operating characteristic (ROC) analysis, or sufficient data to construct contingency tables (true positives, false positives, true negatives, false negatives).

Study Designs: Cross-sectional diagnostic accuracy studies, prospective or retrospective clinical studies, randomized clinical trials, and comparative observational studies.

Language: Articles published in English.

### Exclusion criteria

#### Case reports or case series

Reviews, editorials, conference abstracts, and expert opinions.

#### Animal studies or *in vitro* studies

Studies without clearly reported diagnostic outcomes.

Studies involving adults only or populations where pediatric data could not be separated.

### Study selection

Two reviewers (PS and NJ) independently conducted the literature search across all databases. Retrieved records were compiled into a master reference list, and duplicates were removed using reference management software. Prior to screening, calibration was performed on a subset of studies to ensure consistency in the application of eligibility criteria.

**Figure 1:**
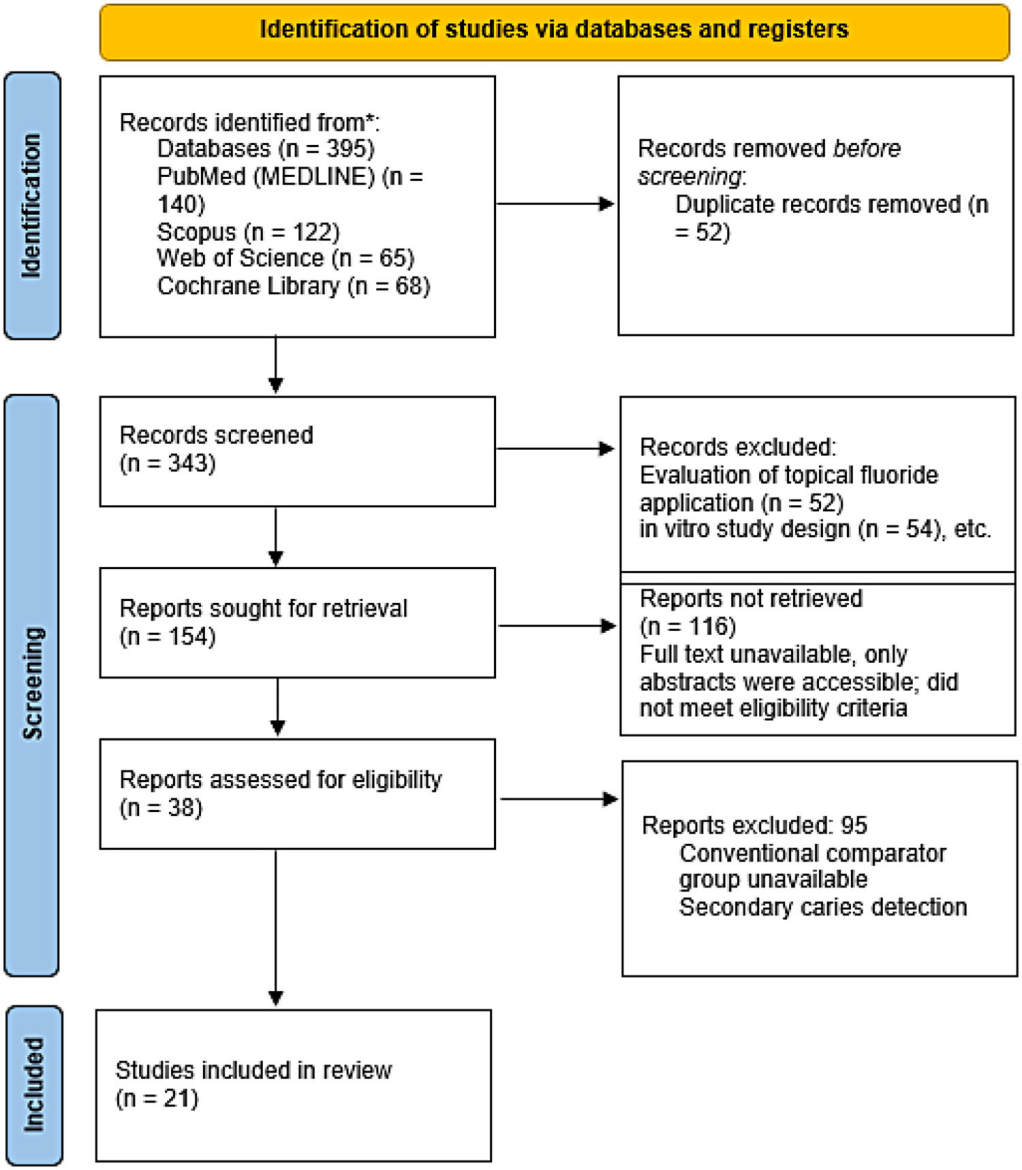
PRISMA flow diagram for study selection.

Titles and abstracts were screened independently by both reviewers to identify potentially relevant studies. Full-text articles of eligible studies were subsequently retrieved and assessed independently against the inclusion criteria. Reasons for exclusion at the full-text stage were recorded and are presented in the PRISMA flow diagram (Figure 1).

Disagreements at any stage were resolved through discussion, and when consensus could not be reached, a third reviewer (SB) was consulted.

### Data extraction

Data extraction was performed independently by two reviewers (PS and NJ) using a standardized, pre-piloted data extraction form. Any discrepancies in extracted data were resolved through discussion and consensus.

The following information was collected and tabulated:

- Author and year of publication
- Study design and clinical setting
- Sample size and participant age range
- Dentition type (primary, permanent, or mixed)
- Type of laser fluorescence device and diagnostic thresholds
- Lesion type and surface examined
- Comparator methods
- Reference standard used
- Examiner calibration and blinding (when reported)
- Diagnostic accuracy outcomes (sensitivity, specificity, predictive values)
- Data required to construct contingency tables (true positives, false positives, true negatives, false negatives)

Where studies reported multiple thresholds or examiners, clinically relevant thresholds or a single examiner dataset were selected to maintain statistical independence.

The characteristics of the included studies are summarized in Table 2.

**Table 2:** Characteristics of included articles.

S. No.	Study (Author, Year)	Teeth/Surfaces (N)	Dentition	Index test (full name)	Comparator/Reference standard (combined)	Lesion type
1	Sheehy et al. (11)	170 permanent molars	Permanent	DIAGNOdent laser fluorescence device (KaVo, Biberach, Germany)	Visual clinical examination using epidemiological diagnostic criteria	Occlusal caries
2	Pinelli et al. (12)	220 smooth surfaces	Primary	DIAGNOdent laser fluorescence device (KaVo, Germany)	Visual clinical examination assessing lesion activity status	Free smooth surface white-spot lesions
3	Anttonen et al. (13)	423 permanent molars + 315 primary molars	Mixed	DIAGNOdent laser fluorescence device (KaVo, Germany)	Visual clinical examination with longitudinal follow-up for caries progression	Fissure caries
4	Costa et al. (17)	264 teeth (199 permanent, 65 primary)	Mixed	DIAGNOdent laser fluorescence device (KaVo—Biberach, Germany)	Visual examination and radiographic examination with operative validation	Occlusal caries
5	Toraman Alkurt et al. (18)	112 permanent molars	Permanent	DIAGNOdent laser fluorescence device (KaVo, Germany)	Visual clinical examination and bitewing radiography with operative validation	Occlusal caries
6	Kavvadia et al. (19)	120 primary molars	Primary	DIAGNOdent laser fluorescence device (KaVo, Germany)	Visual examination with operative validation after cavity opening	Occlusal caries
7	Costa et al. (20)	80 primary molars	Primary	DIAGNOdent laser fluorescence device (KaVo, Germany)	Visual clinical examination with operative validation	Non-cavitated occlusal dentine caries
8	Kühnisch et al. (21)	632 permanent molars	Permanent	DIAGNOdent laser fluorescence device (KaVo, Germany)	WHO criteria and International Caries Detection and Assessment System II with clinical validation	Occlusal caries
9	Novaes et al. (22)	621 approximal surfaces	Primary	DIAGNOdent pen 2,190 (KaVo, Biberach, Germany)	Visual inspection and bitewing radiography with temporary tooth separation using orthodontic elastics as reference standard	Approximal caries
10	Duruturk et al. (23)	104 permanent first molars	Permanent	DIAGNOdent laser fluorescence device (KaVo, Germany)	Visual examination with operative validation	Non-cavitated occlusal caries
11	Souza et al. (24)	79 primary molars	Primary	DIAGNOdent 2,095, DIAGNOdent pen 2,190, VistaProof fluorescence camera	International Caries Detection and Assessment System and bitewing radiography with histological validation	Occlusal caries
12	Çınar et al. (25)	80 primary molars	Primary	DIAGNOdent and DIAGNOdent pen (KaVo, Germany)	Visual examination with histological validation	Occlusal caries
13	Teo et al. (26)	102 primary molars	Primary	DIAGNOdent pen (KaVo, Germany)	International Caries Detection and Assessment System and CarieScan PRO with Downer histological criteria	Occlusal caries
14	Mepparambath et al. (27)	101 primary molars/169 proximal surfaces	Primary	DIAGNOdent laser fluorescence device (KaVo, Germany)	Bitewing radiography with clinical validation	Proximal caries
15	Bussaneli et al. (28)	377 proximal surfaces	Primary	DIAGNOdent pen (KaVo, Germany)	Visual inspection and bitewing radiography with temporary tooth separation	Proximal caries
16	Goswami et al. (29)	176 teeth	Mixed	DIAGNOdent laser fluorescence device (KaVo, Germany)	WHO criteria and International Caries Detection and Assessment System II with clinical validation	Cavitated and non-cavitated caries
17	Kockanat et al. (30)	120 primary molars	Primary	DIAGNOdent pen (KaVo, Germany)	ICDAS II, radiographic examination, CarieScan PRO and SoproLife camera with Downer histological criteria	Occlusal caries
18	Novaes et al. (31)	485 primary molars	Primary	DIAGNOdent pen and fluorescence camera methods	Visual examination with clinical validation of lesion activity status	Occlusal lesion activity
19	Moriyama et al. (32)	834 proximal surfaces	Permanent	DIAGNOdent pen (KaVo, Germany)	Visual inspection and radiographic examination with temporary tooth separation as reference standard	Proximal caries
20	Monea et al. (33)	180 molars	Permanent	DIAGNOdent laser fluorescence device (KaVo, Germany)	Visual inspection using International Caries Detection and Assessment System II	Early pit-and-fissure caries
21	Yehua et al. (34)	120 molars	Permanent	DIAGNOdent pen (KaVo, Germany)	International Caries Detection and Assessment System II with histological validation	Initial occlusal caries

### Reference standards

The diagnostic accuracy results of included studies were interpreted according to the reference standards used for disease verification. Eligible reference standards included:

- Visual examination, with or without standardized criteria such as the International Caries Detection and Assessment System (ICDAS), or combinations of visual and tactile examination
- Bitewing radiography
- Combination of visual–tactile examination and bitewing radiography
- Histological validation of tooth samples or operative validation where available

Given the variability in verification methods across studies, results were interpreted with consideration of the potential influence of different reference standards on diagnostic accuracy estimates. The use of multiple reference standards introduces the possibility of differential verification bias, incorporation bias, and variability in disease classification thresholds. Therefore, findings were stratified and discussed according to the reference standard used whenever possible.

### Lesion threshold definitions

Lesion thresholds were standardized across studies where possible according to commonly used diagnostic classifications. Early enamel or non-cavitated lesions were considered equivalent to the D1 threshold and generally corresponded to International Caries Detection and Assessment System (ICDAS) (16) scores 1–2 or radiographic enamel lesions without dentinal involvement. Dentinal or cavitated lesions were considered equivalent to the D3 threshold and corresponded to ICDAS scores ≥3, radiographic dentinal radiolucency, or operative validation indicating dentin involvement.

Proximal lesions were defined based on clinical or radiographic detection on interproximal tooth surfaces, with some studies incorporating temporary tooth separation as a reference standard. Where studies reported device-specific numerical thresholds (e.g., DIAGNOdent readings), manufacturer-recommended or study-defined cut-off values were used. Variations in thresholds across studies were considered during data synthesis and interpretation of diagnostic accuracy outcomes.

### Risk of bias assessment

The methodological quality of the included studies was assessed using the Quality Assessment of Diagnostic Accuracy Studies-2 (QUADAS-2) (35) tool. QUADAS-2 evaluates risk of bias across four domains: (1) patient selection, (2) index test, (3) reference standard, and (4) flow and timing, and also assesses concerns regarding applicability in the first three domains.

Two reviewers (PS and NJ) independently performed the risk-of-bias assessment following calibration using the QUADAS-2 guidance document. Signalling questions were tailored to the review question, particularly with respect to lesion thresholds, blinding of index test interpretation, and appropriateness of the reference standard (e.g., ICDAS criteria, radiographic examination). Disagreements were resolved through discussion, with consultation from a third reviewer (SB) when necessary.

**Figure 2:**
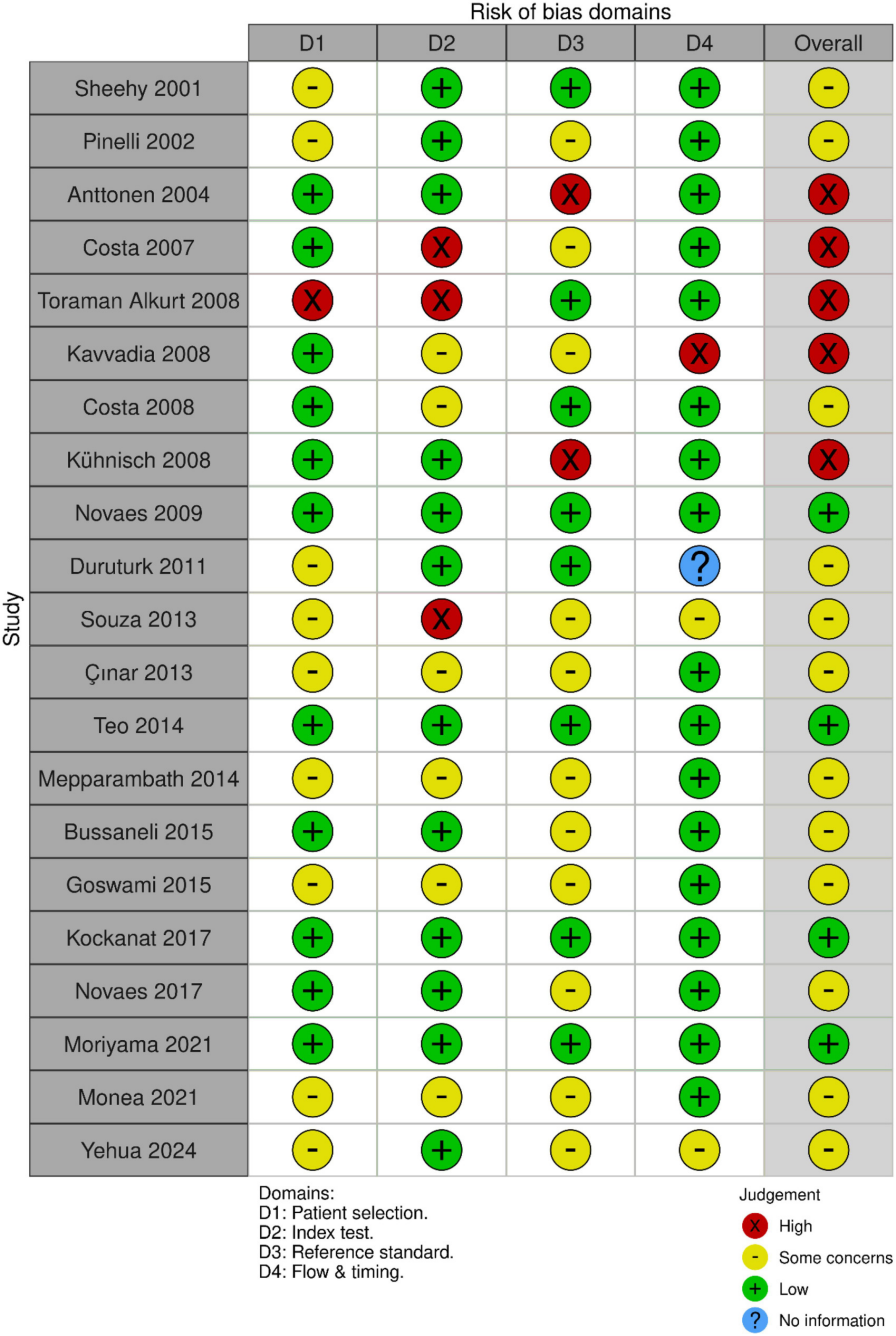
Quality of included studies assessed with QUADAS-2 tool.

Each domain was judged as having low, high, or unclear risk of bias. The results are presented as a traffic-light plot generated using the Robvis tool (36) (Figure 2).

The risk-of-bias assessments were considered during data synthesis and interpretation of findings, particularly when evaluating variability in diagnostic performance across studies.

## Results

### Study selection

The PRISMA flow diagram illustrating the study selection process is presented in Figure 1.

The initial database search identified 395 potentially relevant records. After removal of duplicates, 343 articles remained for title and abstract screening. During the screening process, studies were excluded for the following reasons: evaluation of topical fluoride application (*n* = 52), sealant-related studies (*n* = 11), detection of subgingival calculus (*n* = 1), patient perception studies (*n* = 3), composite resin evaluation (*n* = 2), *in vitro* study design (*n* = 54), examiner comfort evaluation (*n* = 2), methodological evaluation studies (*n* = 7), web-based sources (*n* = 13), calibration technique reports (*n* = 1), endodontic applications (*n* = 1), systematic reviews (*n* = 4), adult-only populations (*n* = 10), light-induced fluorescence studies not involving laser fluorescence (*n* = 3), impedance spectroscopy studies (*n* = 1), non-English language article (*n* = 1), inaccessible full texts (*n* = 17), registered clinical trials with unknown status (*n* = 3), narrative review (*n* = 1), case report (*n* = 1), and microbial studies (*n* = 1).

Following application of the eligibility criteria, 21 studies were included in the final qualitative synthesis.

### Risk of bias assessment

The methodological quality of the included studies is summarised in Figure 2. Only four studies (Novaes 2009; Teo 2014; Kockanat 2017; Moriyama 2021) were judged to have low risk of bias across all QUADAS-2 domains, whereas the majority of studies presented either some concerns or high risk of bias in at least one domain.

The most frequent concerns were observed in the patient selection (Domain 1) and reference standard (Domain 3) domains. Several studies employed convenience sampling or did not clearly describe participant recruitment methods, introducing potential spectrum bias. This may have influenced diagnostic accuracy estimates by including lesions with more obvious clinical presentation. High risk of bias in the reference standard domain was noted in studies that relied solely on visual criteria or radiographic assessment without histological validation. Variability in lesion thresholds (e.g., enamel vs. dentinal caries) may have introduced differential verification bias and contributed to heterogeneity in reported sensitivity and specificity.

Index test bias (Domain 2) was less common but was identified in studies where blinding of laser fluorescence readings to reference standard results was not clearly reported. The flow and timing domain (Domain 4) generally demonstrated low risk; however, incomplete reporting in some studies limited certainty regarding verification procedures.

Overall, studies with higher risk of bias in patient selection or reference standard domains tended to report higher sensitivity estimates, suggesting possible overestimation of diagnostic performance. These methodological limitations were considered when interpreting the pooled results.

### Assessment of heterogeneity and subgroup analyses

Clinical and methodological heterogeneity were anticipated due to differences in device type (DIAGNOdent vs. DIAGNOdent Pen), diagnostic threshold cut-off values, lesion location (occlusal, proximal, or smooth surfaces), dentition (primary vs. permanent teeth), and reference standards used (visual criteria, radiographic examination, or combined methods).

Heterogeneity was assessed qualitatively by comparing study characteristics, risk-of-bias domains, and diagnostic thresholds across studies. Where quantitative synthesis was performed, heterogeneity was further evaluated using hierarchical models and visual inspection of study estimates in receiver operating characteristic (ROC) space. Forest plots were used to examine variability in sensitivity and specificity estimates across studies.

Subgroup analyses were pre-specified to explore potential sources of heterogeneity based on the following factors:

- **Device type:** DIAGNOdent vs. DIAGNOdent Pen
- **Lesion location:** Occlusal vs. proximal vs. smooth surfaces
- **Dentition:** Primary vs. permanent teeth
- **Lesion threshold:** Enamel/non-cavitated vs. dentinal/cavitated lesions
- **Reference standard type:** Visual examination alone vs. combined visual and radiographic assessment

Variability in diagnostic thresholds across studies was also considered as a potential source of heterogeneity and threshold effects were explored during interpretation of findings.

### Statistical analysis and data synthesis

#### Quantitative synthesis

Quantitative synthesis was undertaken for studies that provided sufficient diagnostic accuracy data and were considered clinically and methodologically comparable. Studies were grouped according to lesion threshold, dentition type, and reference standard to minimise heterogeneity. A hierarchical approach using bivariate random-effects and hierarchical summary receiver operating characteristic (HSROC) models was applied to jointly estimate pooled sensitivity and specificity while accounting for between-study variability and potential threshold effects.

Statistical analyses were conducted using the mada package in R software, version 4.5.2. (R Foundation for Statistical Computing, Vienna, Austria).

### Meta-analysis at D1 threshold (enamel and early dentinal lesions)

A meta-analysis was conducted for studies evaluating laser fluorescence devices at the D1 diagnostic threshold (enamel and early dentinal caries). Eight studies (Novaes (22); Souza (24); Teo (26); Çınar (25); Kockanat (30); Yehua (34); Goswami (29); Costa (17) providing sufficient data were included in the hierarchical analysis. Using a bivariate random-effects model, the pooled sensitivity was 0.84 (95% CI 0.80–0.87) and the pooled specificity was 0.77 (95% CI 0.67–0.84).

**Figure 3:**
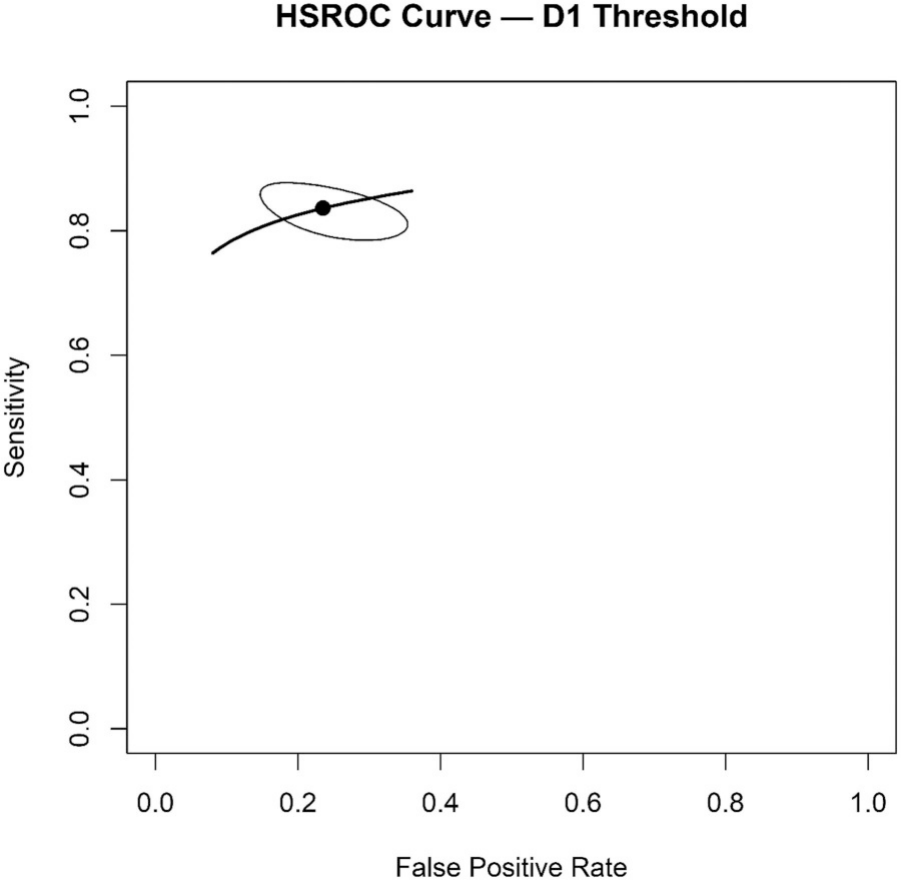
HSROC curve at D1 threshold for enamel and early dentinal lesions.

The HSROC curve demonstrated good overall diagnostic performance, with an area under the curve (AUC) of 0.86 (Figure 3). Between-study heterogeneity was low after adjustment for sample size effects, with adjusted *I*^2^ estimates ranging from approximately 0.8%–4.1%, suggesting relatively consistent diagnostic performance across included studies.

**Figure 4:**
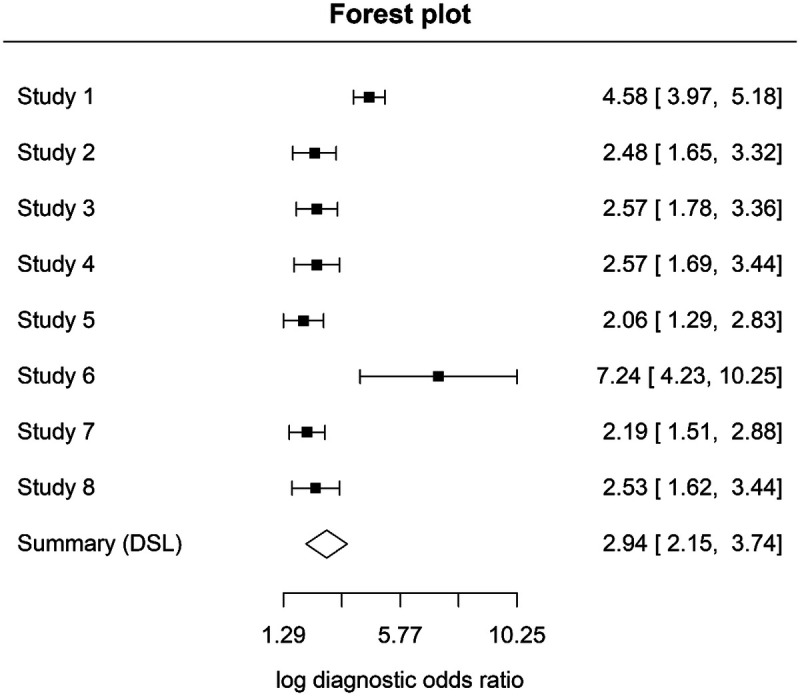
Sensitivity forest plot at D1 threshold.

**Figure 5:**
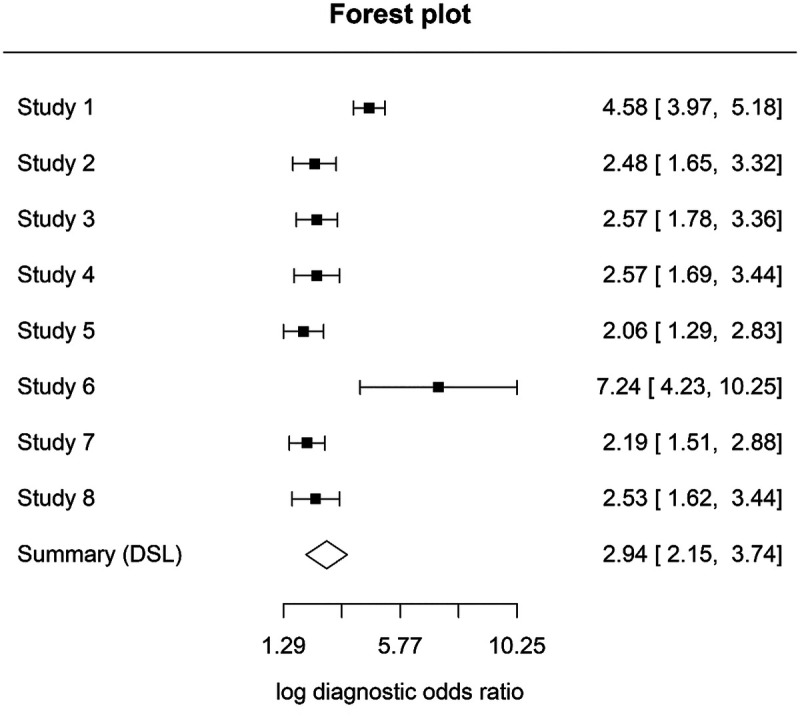
Specificity forest plot at D1 threshold.

Forest plots illustrating study-level sensitivity and specificity estimates are presented in Figures 4, 5.

### Meta-analysis at D3 threshold (dentinal lesions)

A quantitative synthesis was also performed for studies evaluating laser fluorescence devices at the D3 diagnostic threshold (dentinal caries). Ten studies (Sheehy (11); Anttonen (13); Toraman Alkurt (18); Kavvadia (19); Kühnisch (21); Duruturk (23); Mepparambath (27); Bussaneli (28); Moriyama (32); Monea (33) were included in the hierarchical analysis. Using a bivariate random-effects model, the pooled sensitivity was 0.81 (95% CI 0.77–0.85) and the pooled specificity was 0.89 (95% CI 0.84–0.92).

**Figure 6:**
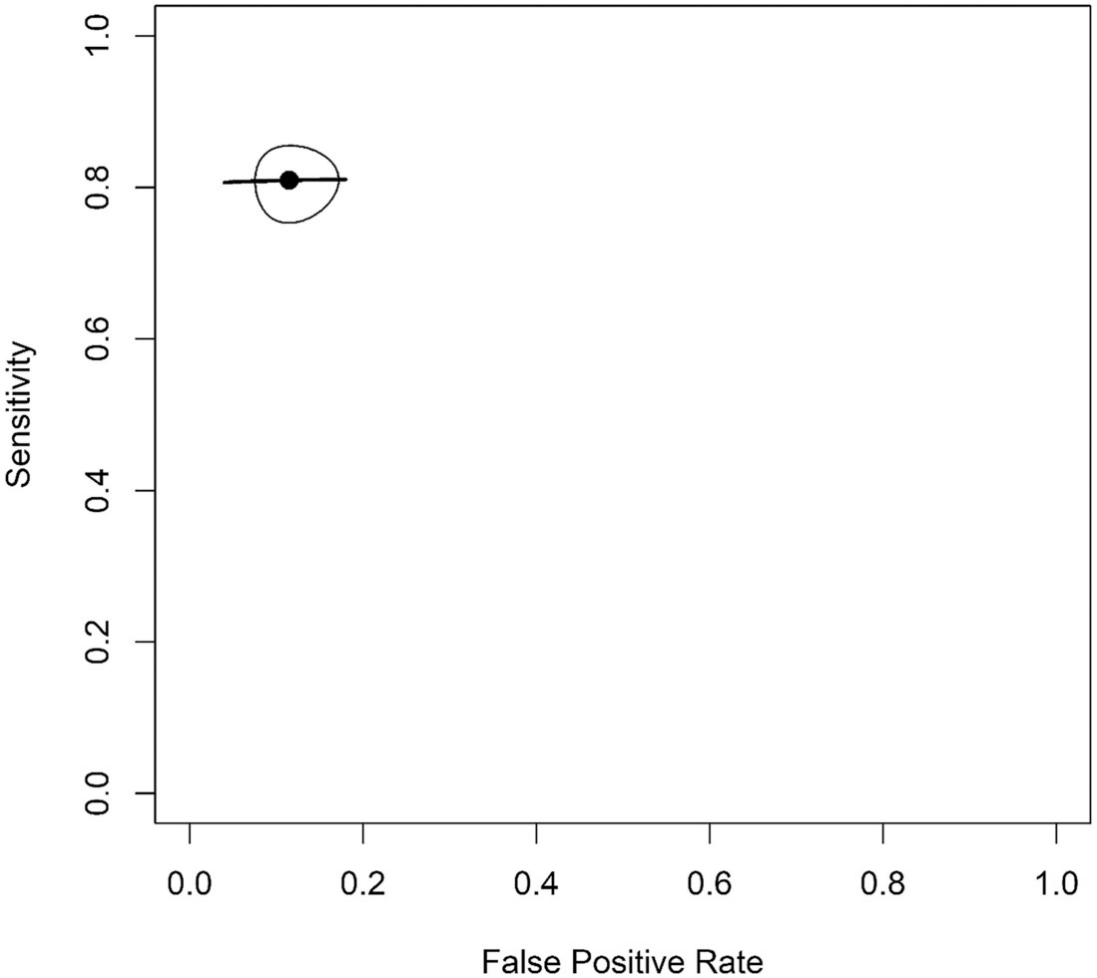
HSROC curve at D1 threshold for dentinal lesions.

The HSROC curve indicated good diagnostic performance, with an area under the curve (AUC) of 0.81 (Figure 6). Between-study heterogeneity was negligible, with adjusted I² estimates close to 0%, indicating highly consistent results across studies.

**Figure 7:**
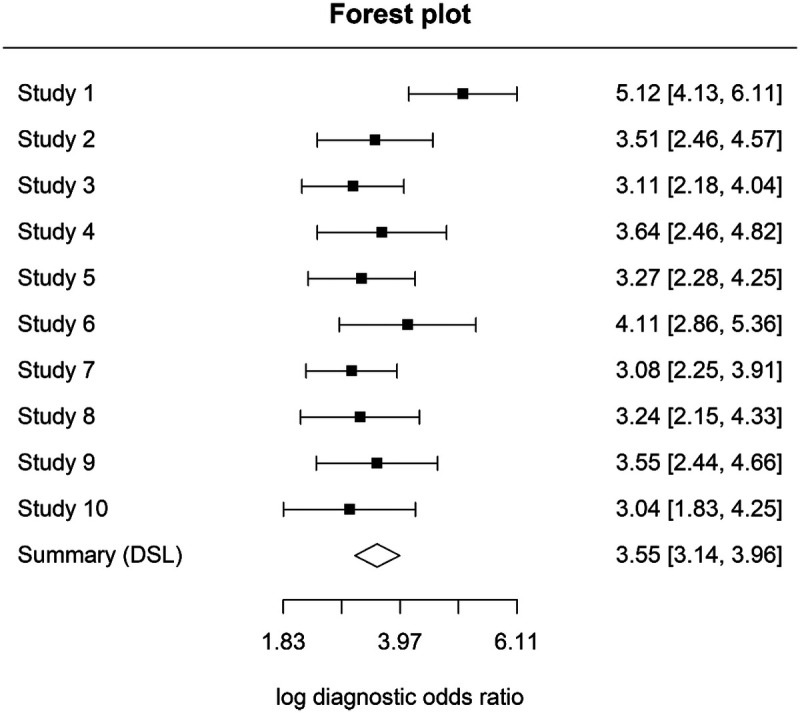
Sensitivity forest plot at D3 threshold.

**Figure 8:**
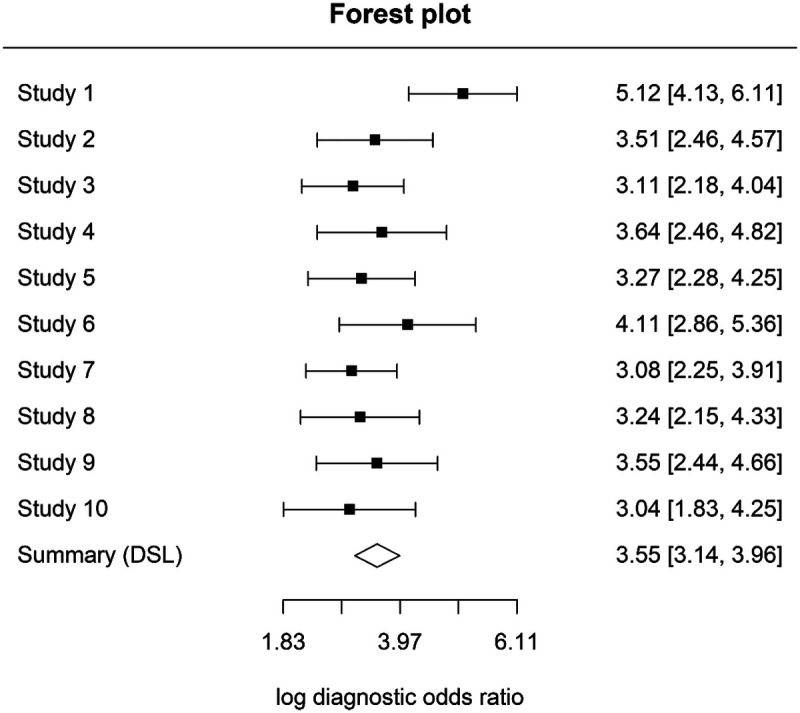
Specificity forest plot at D3 threshold.

Forest plots illustrating variability in sensitivity and specificity across studies are presented in Figures 7, 8.

A summary of the quantitative analysis is presented in Table 3.

**Table 3:** Summary of quantitative findings.

**Threshold**	**Studies (n)**	**Sensitivity (95% CI)**	**Specificity (95% CI)**	**AUC**
D1	8	0.84 (0.80–0.87)	0.77 (0.67–0.84)	0.86
D3	10	0.81 (0.77–0.85)	0.89 (0.84–0.92)	0.81

## Discussion

This systematic review evaluated the diagnostic accuracy of laser fluorescence–based caries detection methods compared with conventional diagnostic approaches in pediatric populations. The findings suggest that laser fluorescence devices demonstrate relatively high sensitivity for detecting early enamel lesions, particularly on occlusal and smooth surfaces, although specificity varies depending on lesion threshold, dentition type, and reference standard used (37, 38).

Threshold selection was an important source of variability across included studies. Lower diagnostic thresholds, particularly for enamel lesions, tended to increase sensitivity but reduce specificity, contributing to higher false-positive rates (39). This effect is consistent with the biological mechanism of fluorescence-based detection, where signals may be generated from non-carious factors such as staining, plaque, or developmental defects (7). Conversely, higher thresholds corresponding to dentinal involvement demonstrated improved specificity and more consistent diagnostic performance across studies. These findings highlight the importance of standardized diagnostic cut-off values when interpreting laser fluorescence readings in clinical practice.

The quantitative synthesis further supported these observations, with pooled sensitivity of 0.84 at the D1 threshold and 0.81 at the D3 threshold, indicating good ability of laser fluorescence devices to identify carious lesions. However, specificity was more variable at early lesion thresholds, reflecting a greater potential for false-positive readings (37).

The higher sensitivity of laser fluorescence devices for early lesions can be explained by their detection mechanism, which relies on fluorescence emitted by bacterial metabolites within demineralized tooth structure (40). This may allow identification of subsurface changes before cavitation becomes clinically visible. However, the increased sensitivity was often accompanied by reduced specificity, particularly at enamel-level thresholds. Fluorescence signals may be influenced by plaque accumulation, staining, calculus, and developmental enamel defects, which can lead to overestimation of lesion severity if readings are interpreted without clinical correlation (7, 41). These findings reinforce the importance of using laser fluorescence as an adjunct rather than a standalone diagnostic tool.

Diagnostic performance also varied according to lesion location. For proximal surfaces, several included studies reported performance comparable to bitewing radiography at dentinal thresholds, whereas detection of early enamel proximal lesions remained less reliable. Bitewing radiographs therefore continue to play an essential role in identifying proximal cavitation and lesion depth, particularly in clinical decision-making for restorative intervention (42). Conversely, laser fluorescence appeared more useful for occlusal and smooth surface lesions, where radiographic sensitivity is limited (43).

Differences between primary and permanent dentition may also influence fluorescence-based detection. Structural variations, including thinner enamel and higher organic content in primary teeth, may alter fluorescence responses and contribute to differences in diagnostic accuracy across studies (44). These findings highlight the importance of interpreting device readings within the context of dentition type and lesion stage.

The methodological quality of included studies influenced the interpretation of results. Studies with higher risk of bias in the patient selection and reference standard domains tended to report higher sensitivity estimates, suggesting possible overestimation of diagnostic performance. Variability in reference standards, including the use of visual criteria alone, radiographic assessment, or histological validation, introduced potential differential verification and incorporation bias (45). These factors likely contributed to heterogeneity observed across studies and should be considered when interpreting pooled estimates.

Although most studies were conducted under clinical conditions, the inclusion of histological validation using extracted teeth in some studies may limit direct generalizability to routine clinical practice.

Beyond diagnostic accuracy, practical considerations influence the clinical adoption of laser fluorescence devices in pediatric dentistry. These devices offer several advantages, including non-invasive assessment, absence of ionizing radiation, and immediate numerical feedback, which may enhance patient communication and motivation for preventive care. Examination time is generally short once the operator is familiar with device handling; however, appropriate training and calibration are required to ensure reliable readings and to interpret results within the clinical context (46). In pediatric patients, cooperation levels may influence measurement accuracy, particularly because adequate tooth cleaning and drying are necessary before assessment. The presence of plaque, calculus, staining, or developmental enamel defects can produce elevated fluorescence values and increase the likelihood of false-positive findings, emphasizing the importance of proper surface preparation and clinical judgment (7).

Cost considerations also affect implementation, as fluorescence devices represent an additional financial investment compared with conventional visual examination. Nevertheless, their potential role in early lesion detection and monitoring may support minimally invasive treatment strategies and reduce the need for restorative interventions over time. Infection control is another relevant factor in pediatric settings, requiring appropriate barrier protection and disinfection of device tips between patients according to manufacturer recommendations (47). Overall, laser fluorescence devices are best considered adjunctive tools that complement conventional visual and radiographic assessment rather than replacements, particularly in pediatric clinical environments where cooperation, lesion activity assessment, and preventive decision-making are central to care.

Overall, the evidence supports the use of laser fluorescence devices as adjunctive tools for early caries detection and monitoring, particularly in preventive and minimally invasive management strategies. However, conventional visual examination and radiographic assessment remain essential for determining lesion depth and cavitation status. Clinical decisions should therefore integrate fluorescence readings with comprehensive clinical assessment rather than relying solely on device measurements.

### Limitations

Several limitations should be considered when interpreting the findings of this review. Although quantitative synthesis was performed for selected diagnostic thresholds, substantial clinical and methodological heterogeneity was present across included studies. Variations in lesion classification thresholds, dentition type, diagnostic protocols, and reference standards may have influenced pooled estimates and limited direct comparability between studies.

Many studies relied on visual or radiographic reference standards rather than histological validation, introducing the potential for differential verification and incorporation bias. Differences in examiner calibration, device settings, and surface preparation procedures (e.g., cleaning and drying protocols) may also have contributed to variability in reported diagnostic accuracy.

Additionally, several studies reported outcomes per tooth or per surface rather than per patient, introducing potential clustering effects that were not consistently accounted for in primary analyses. This may have influenced confidence intervals and the precision of diagnostic estimates. Finally, the relatively small number of studies within subgroup analyses limited the ability to perform more detailed stratified meta-analyses according to device type, lesion location, and reference standard.

### Clinical implications and future directions

From a clinical perspective, laser fluorescence devices may enhance early caries detection in children when used as adjuncts to conventional visual and radiographic examination. Their non-invasive nature, rapid feedback, and potential to aid monitoring of non-cavitated lesions make them particularly useful in preventive and minimally invasive pediatric dentistry. However, variability in specificity and susceptibility to confounding factors such as plaque, staining, and developmental defects indicate that fluorescence readings should be interpreted within the context of comprehensive clinical assessment.

Future research should focus on the development of standardized diagnostic thresholds, improved calibration protocols, and consistent reference standards to reduce heterogeneity across studies. Longitudinal investigations evaluating lesion progression and treatment outcomes are also needed to determine the clinical impact of fluorescence-based detection technologies. Additionally, well-designed comparative studies assessing newer fluorescence devices under standardized clinical conditions would further clarify their role in pediatric caries management.

## Conclusion

Laser fluorescence–based devices demonstrate good sensitivity for the detection of early carious lesions in pediatric populations, particularly at enamel-level thresholds and on occlusal or smooth surfaces. However, specificity varies considerably depending on lesion stage, dentition type, diagnostic thresholds, and reference standards used. Quantitative synthesis indicated favourable diagnostic performance at both D1 and D3 thresholds, although methodological heterogeneity and risk-of-bias concerns across studies warrant cautious interpretation.

Laser fluorescence devices should therefore be considered adjunctive tools that may support early detection and monitoring of caries progression rather than replacements for conventional visual examination and radiographic assessment. Bitewing radiography remains essential for evaluating proximal lesion depth and cavitation status in clinical decision-making. Future research using standardized diagnostic thresholds, consistent reference standards, and longitudinal clinical outcomes is required to clarify the optimal role of fluorescence-based technologies in pediatric caries management.

## Data Availability

The datasets presented in this study can be found in online repositories. The names of the repository/repositories and accession number(s) can be found below: https://www.crd.york.ac.uk/PROSPERO/view/CRD420251274327.
